# Dr. Geo. Sutton on Trichinosis

**Published:** 1875-09

**Authors:** 


* * * Dr. George Sutton, of Aurora, Indiana, read a lengthy manuscript on Trichinosis, setting forth the great prevalence of this disease, the extent of its fatality, and methods of treatment. A number of cases were cited showing the symptoms of trichinosis and its progress in the system. It was a very interesting and profitable paper. The probability is that the disease is of great antiquity, causing the ancient Jew and Turk to prohibit the use of pork. From microscopical examination of pork brought to his office, he was satisfied that of the pork killed in the northeastern portion of Indiana, a large portion was affected with the parasite. He thought that when the fact becomes more generally known that so large a per cent. is affected with trichina, and capable of producing so loathsome a disease, it will have an influence upon one of the principal productions of the West—affecting the agriculturists, and even to some extent the commerce of the country. He cited a large number of cases to show that a very large number of cases of chronic diarrhœa, chronic dysentery and chronic enteritis was the result of trichina. He showed from statistics the large number of persons that died from symptoms that were obviously, in his opinion, those of trichinosis. Of the cases he attended afflicted with this disease, thirty per cent. proved fatal. The doctor aimed to show that it was much more prevalent than was generally supposed, and that such was the tenacity of life of these insects that it required a great deal of cooking to kill them in the pork; also, that trichina do not necessarily exist in the muscles to produce disease, but that their presence in the bowels produced symptoms that often proved serious. However, in cases proving fatal they were found in the muscular system in large numbers.—*Indiana Jour. Med.*

